# Meeting the need for effective and standardized neonatology training: a pan-European Master’s Curriculum

**DOI:** 10.1038/s41390-024-03182-8

**Published:** 2024-05-03

**Authors:** Deanna Santoro, Devin A. Zibulsky, Charles C. Roehr, Florian Langhammer, Max Vento, Tomasz Szczapa, J.-C. Fauchère, Gabriel Dimitriou, Heike Rabe, Silke Mader, Luc J. I. Zimmermann, Deirdre M. Murray, Susan Smith, Mike Hall, Manfred Künzel, Sven Wellmann

**Affiliations:** 1 European Society for Paediatric Research (ESPR), Satigny, Switzerland; 2 Department of Paediatrics and Child Health, University College Cork, Cork, Ireland; 3 Independent Researcher, Cork, Ireland; 4 National Perinatal Epidemiology Unit, Oxford Population Health, Medical Sciences Division, University of Oxford, Oxford, UK; 5 Faculty of Health Sciences, University of Bristol, Bristol, UK; 6 Department of Neonatology, University Children’s Hospital Regensburg (KUNO), Hospital St. Hedwig of the Order of St. John, University of Regensburg, Regensburg, Germany; 7 Division of Neonatology, University and Polytechnic Hospital La Fe (HULAFE), Valencia, Spain; 8 II Department of Neonatology, Neonatal Biophysical Monitoring and Cardiopulmonary Therapies Research Unit, Poznan University of Medical Sciences, Poznan, Poland; 9 Newborn Research, Department of Neonatology, University Hospital Zurich, University of Zurich, Zurich, Switzerland; 10 Department of Paediatrics, University of Patras Medical School, Patras, Greece; 11 Brighton and Sussex Medical School, University of Sussex, Brighton, United Kingdom; 12 European Foundation for the Care of Newborn Infants (EFCNI), Munich, Germany; 13 Department of Paediatrics-Neonatology and School for Oncology and Reproduction, Maastricht UMC+, Maastricht, The Netherlands; 14 School of Health Sciences, University of Southampton, Southampton, UK; 15 PHW Business School Bern, Bern, Switzerland

## Abstract

**Abstract:**

Neonatology is a pediatric sub-discipline focused on providing care for newborn infants, including healthy newborns, those born prematurely, and those who present with illnesses or malformations requiring medical care. The European Training Requirements (ETR) in Neonatology provide a framework for standardized quality and recognition of equality of training throughout Europe. The latest ETR version was approved by the Union of European Medical Specialists (UEMS) in April 2021. Here, we present the curriculum of the European School of Neonatology Master of Advanced Studies (ESN MAS), which is based on the ETR in Neonatology and aims to provide a model for effective and standardized training and education in neonatal medicine. We review the history and theory that form the foundation of contemporary medical education and training, provide a literature review on best practices for medical training, pediatric training, and neonatology training specifically, including educational frameworks and evidence-based systems of evaluation. The ESN MAS Curriculum is then evaluated in light of these best practices to define its role in meeting the need for a standardized empirically supported neonatology training curriculum for physicians, and in the future for nurses, to improve the quality of neonatal care for all infants.

**Impact statement:**

- A review of the neonatology training literature was conducted, which concluded that there is a need for standardized neonatology training across international contexts to keep pace with growth in the field and rapidly advancing technology. This article presents the European School of Neonatology Master of Advanced Studies in Neonatology, which is intended to provide a standardized training curriculum for pediatricians and nurses seeking sub-specialization in neonatology. The curriculum is evaluated in light of best practices in medical education, neonatology training, and adult learning theory.

## Introduction

Neonatal care is a growing discipline, which evolved in the 1960s as an independent subspecialty of pediatrics.^1,2^ Neonatology practitioners need extensive knowledge, skills, and expertize to provide care to critically ill term and preterm newborn infants.^3^ Research has shown that neonatology practitioners also need experience performing key skills for effective clinical practice.^4^ As medical knowledge and technology advance, neonatal care must evolve alongside this progress and increasing complexity to achieve successful outcomes for every infant and address the future research needs of neonatology.^5^ The only way to meet this growing and evolving need is through effective neonatal education, which begins with standardized, comprehensive training programs that incorporate empirically supported best practices to ensure that healthcare providers are well-equipped to deliver safe and effective care.^6–9^

In recent years, neonatal care has shifted from being case driven to infant and family centered developmental care (IFCDC), which represents a more robust approach to achieving optimal long-term outcomes.^10^ To train neonatal professionals in this new approach, the European School of Neonatology (ESN) has developed a curriculum that aligns with the European Training Requirements (ETR) in Neonatology, providing a structured and standardized approach to neonatal training and education. The present paper will evaluate the neonatology training curriculum developed by the ESN to determine whether it can fulfill the need for a standardized neonatology training approach for physicians and eventually also for nurses.

According to curriculum theory,^11^ an effective curriculum provides an agreed upon set of definitions for key technical concepts within a field, classifies recent developments within an established knowledge base, and draws upon research-based knowledge to inform practice. The purpose of developing a curriculum is to reach a level of consensus within a field about what trainees should know and be able to do following training. The ESN’s European Curriculum & Evaluation Grid for Training and Assessment in Neonatology provides an in-depth step-by-step guideline of critical competencies for neonatology practitioners based on previous international frameworks and expert consultation. It integrates research-based practices that incorporate the most modern approaches within established knowledge and practices, taking a trainee-centered approach to training for infant and family-centered developmental care. In this way, the ESN curriculum synthesizes predominant theory and practices within the field into a standardized training framework rooted in the best practices of medical education.

### History of medical education and neonatology training

Medical education occupies a unique place in higher education. It extends past traditional university research departments and into hospitals to teach novice practitioners how to care for real patients.^12^ The goal of medical education is to provide society with a consistent supply of medical professionals who put patient care first. This means that the stakes in medical education are high in two separate ways; one is the societal need for producing skilled practitioners and the second is the inherent potential for serious consequences arising from novice practitioners working directly with real patients.

In adapting to the need for practical experience rooted in the latest knowledge within the field, medical education has evolved to incorporate many educational philosophies, conceptual models, and pedagogical practices, which can vary across specialties and individual training settings. However, the predominant approach to medical education combines elements of educational theory, such as experiential learning and reflective practice, with elements of vocational training, such as competency-based evaluation.^12^

By the mid twentieth century, medical training programs in Europe had developed a regimented curriculum in which units were strictly time-limited.^13^ However, this approach faced criticism for being too rigidly structured to allow opportunities for nuance and deeper learning, and for being focused on maintaining the structure at the expense of developing learners’ competencies. As a result of this criticism, competency based medical education (CBME) was introduced which provided a set of guidelines for the skills that trainees must master to complete their training. One challenge faced by European medical educators following the CBME model is that as the body of medical knowledge and technology have grown, so too have the content and duration of medical training programs.^13^ As curricula have been adjusted to fit the need for ever-growing content knowledge within acceptable timelines, there is demand from professionals within the field for international training standards.^14^

To meet the need for a specialized pediatric curriculum in Europe, pediatric training requirements were developed by the European Academy of Paediatrics (EAP), a professional organization of pediatricians, which is the pediatric branch of the European Union of Medical Specialists. The most recent UEMS Training Requirements for the Specialty of Paediatrics were published in 2015.^15^ One of the key goals of this European framework for pediatrics is to create a “common house for pediatrics” in which the many subspecialities of pediatrics can come together.^15^ The authors note that pediatrics as a field has become increasingly specialized, and to bridge differences in resources and scope, pediatric training needs a standardized training approach.

The training framework itself is divided into sections focused on theoretical knowledge and on practical and clinical skills.^15^ This reflects the previously discussed need for medical training to strike a balance between knowledge and practice. The theoretical knowledge section of the framework contains a comprehensive list of pediatric subspecialties, which reflects the increasingly specialized nature of the field. The practical skills section includes a list of observable skills that practitioners must master. These skills are applicable across subspecialties and reflect the use of various medical technologies. By bridging theory and practice, the training framework is broadly applicable across training contexts while also being detailed enough to reflect the key components of theoretical knowledge.

The ETR Neonatology, released in 2021, was developed in collaboration between the pediatrics section of UEMS along with the European Board of Neonatology^16^ (European Society for Paediatric Research, 2021). Previous versions of the training requirements were created in 1998 and in 2007. The training requirements were updated to include the most recent developments in technology and clinical practice, as well as incorporating knowledge and best practices of national training programs that did not fit the previous training framework. The ETR Neonatology emphasizes the modern multidisciplinary team approach to neonatology in which neonatology practitioners are never acting alone, but rather are constantly communicating and collaborating with many other stakeholders, including families, nurses, midwives, other medical professionals, psychologists, and social workers.

The ETR Neonatology places particular emphasis on the end goals and skills expected from neonatology training while also providing details about time frames, accreditation, evaluation, and assessment.^16^ It provides comprehensive instructions for what resources and programs training centers need to provide neonatology training and how novice practitioners are tracked in terms of learning and progression of skills. The ETR provides a thorough overview of knowledge within the field of neonatology and of the skills and practices that neonatologists need. The aims in creating the ETR Neonatology are to harmonize neonatology training across European countries, establish clear standards for skills and knowledge, and facilitate the development of neonatology care centers, with the underlying goal of improving health care for neonates.^16^ Neonatal practitioners with certified training according to the ETR Neonatology can use their pan-European certificate of training to move freely within Europe to practice Neonatology at an accredited expert level.

## Theories of teaching and learning

An effective neonatal training curriculum must incorporate the neonatology training requirements into a training approach based on theoretically developed and empirically supported best practices for training adult learners. Green and Ellis^17^ examined adult learning theory to determine its most relevant applications to medical education. The four key points identified for adult learners were: 1) knowing why they were learning the particular content, 2) having a sense of self-direction, 3) using their personal experience, 4) and seeing applications to real-world situations. These characteristics can form the basis for a broadly applicable framework for effective adult education in a medical context.

The Association for Medical Education in Europe (AMEE) has developed a guide for using adult learning theory for teaching and learning in medical education.^18^ One key learning theory that can be linked to medical training is experiential learning theory.^19^ In this theory, a learner has an experience and then goes through a cycle in which they reflect on their experience, develop concepts and generalizations based on their experience, and then consolidate their understanding to apply in new situations. Within medical education, when a learner/trainee sees a patient or attends a lecture, they compare what they are seeing to their prior knowledge and experience to refine their understanding.^18^ Within an adult learning theory informed approach to medical training, the instructor is responsible for providing context for learning, providing relevant learning experiences at an appropriate level, encouraging reflection, providing feedback, and providing opportunities for rehearsal and application of new knowledge. To be effective, a neonatology training curriculum must provide learners with opportunities to engage in the experiential learning process within their training so that they can apply it to their neonatology knowledge and skills.

## Effective approaches to neonatal education and training

A successful neonatology training program will combine these reflective adult learning practices within the best practices of the field itself to create an integrated approach to care throughout the training experience. One of these best practices is IFCDC, which is a recently elaborated approach to neonatal care that emphasizes the role of parental nurturing and the creation of an age-appropriate healing environment.^20^ This approach has become a primary focus in neonatology practice. It includes the key roles of parental physical presence with the child, breastfeeding and nutrition, communicating health knowledge to parents, and providing support and mental health support. When parents of patients are involved in neonatal clinical care and research, care providers reported that they improved their work and the parents themselves reported largely positive experiences.^10^ The IFCDC approach expands from an earlier perspective that the neonatologist was the holder of knowledge and delivered the care, to focus on the key roles of the family and the broader context.

Within the IFCDC model, a successful neonatology training program can also incorporate elements of error management and quality assurance. While quality can be a difficult concept to measure, one way in which it can be assessed is through the minimization of errors.^21^ Error management theory focuses on the objective measurement of errors, which are defined as deviations from standard operating practices. It emphasizes the importance of tracking and measuring errors to evaluate the quality of training and skills of practitioners. Tracking errors enables an organization to create a feedback loop through which lessons are learned by stakeholders at all levels of the organization.^21^ Error management can help prevent the same errors from being repeated and can be used to create a quality assurance framework for a training program.

Another approach that emphasizes a broader team-based perspective of neonatal practice is Crew Resource Management (CRM). Originally developed for airplane flight crews, CRM focuses on human factors, effective teamwork, and communication and has been applied across a range of healthcare settings.^22^ Using a CRM approach, each member of the medical team and the family have a role in patient care and treatment. CRM training can be brief and can be adapted to a specific medical context or as a broader part of cultural change within an organization.^22^

One area of neonatology training in which the approaches described above can be applied is during simulation training. Simulation training puts the novice practitioner in a mock clinical situation to practice and display the skills they are learning. Simulator training is a widely used component of neonatology training, used to teach skills such as resuscitation techniques, and has been associated with positive learning outcomes.^23^ However, it is essential that simulation training accurately reflects the clinical situation and patient physiology, or it can lead to learning improper techniques.^24^

## The ESN MAS in neonatology curricular approach

The ESN has developed a training framework for neonatal education for its MAS program, known as the European Curriculum & Evaluation Grid for Training and Assessment in Neonatology (Supplement file). This curriculum provides a comprehensive and interprofessional training framework for neonatal doctors and in the future also advanced nurse practitioners involved in neonatal care. The curriculum is designed to align with the ETR Neonatology.^16^ The curriculum is divided into 13 modules, based on previous conceptual work from colleagues, who performed a Delphi process and yielded 13 EPAs with which to assess capability to practice clinical neonatology.^25^

Each ESN MAS module focuses on specific competencies and skills that are essential for providing quality care to neonates (see Table 1), which are derived from the ETR Neonatology. In terms of further development, the EPAs on neonatal transport and transition of care,^25^ are now combined into module 12 of the ESN MAS, while module 13 focuses on IFCDC and related key competencies and skills.

**Table 1:** ESN MAS curriculum modules.

MAS training module	Details
1) Resuscitation and stabilization	Institutes and leads neonatal resuscitation and stabilization according to the latest guidelines. Performs antenatal counseling, communicates with parents.
2) The very and extremely preterm infant	Performs transition at the threshold of viability and recognizes the risk factors for morbidity and mortality of this highly vulnerable group. Supports opportunities of family-centered empowerment care.
3) The moderate and late preterm infant	Performs transition and institutes all diagnostic and therapeutic measures in healthy and critically compromised preterm infants
4) Cardiorespiratory failure	Manages emergencies resulting from cardiac or respiratory causes. Understands the pathophysiology of neonatal circulation and neonatal breathing and recognizes specific diseases
5) Life-threatening infection	Knows principles of pathophysiology, pathogenesis and clinical presentation of neonatal infections and their management
6) Brain-injured newborn	Performs clinical examinations, assesses integrity of the brain using neurophysiologic and imaging tools, initiates therapy and recommends post discharge care and follow-ups
7) Nutrition of the critically ill newborn	Cares for neonates with gastrointestinal emergencies and collaborates interprofessionally with specialists. Knows and adheres to the principles of lactation, breastfeeding and kangaroo-mother care.
8) Surgical problems	Provides care to patients in the NICU with surgical problems in collaboration with pediatric and subspecialty surgeons
9) Single system diseases	Manages patients with acute common single system diseases in an inpatient setting.
10) Bronchopulmonary dysplasia	Cares for infants at risk and with established bronchopulmonary dysplasia.
11) Difficult care conference	Leads complex cases multi-disciplinary care conferences, including discontinuation of life support
12) Neonatal transport and patient flow management and transition of care	Coordinates transport of critically ill newborns, knows principles of neonatal ward management, and performs transition of care: routine sign-out, change of service, discharge.
13) Infant and family centered developmental care	Enables a supportive environment of care, engages and empowers parents as primary caregivers.

There are several components of the ESN MAS Curriculum that are not explicitly addressed in the ETR Neonatology, including sections delineated by stages of preterm birth, surgical problems, single system diseases, and bronchopulmonary problems. The inclusion of these modules within the curriculum reflects the highly specialized nature of neonatology training as developed by Parker et al.^25^ The ESN MAS curriculum has given special focus to these areas, which can fall under multiple areas of the ETR Neonatology competencies and principles. While all sections of the ETR neonatology are covered within the ESN MAS curriculum, some ETR topics are not stand-alone modules within the ESN MAS but these concepts, including ward organization, communication, counseling, and ethical considerations, are integrated into the 13 modules of the ESN MAS curriculum.

The curriculum includes an explicit emphasis on IFCDC, which draws upon the multi-disciplinary and collaborative nature of neonatal care. The inclusion of IFCDC as a major component of the training program will link theoretical knowledge of neonatology to the real-world context in which it is practiced by bringing focus to the role of parents, legal guardians, and responsible care providers. Consequently, IFCDC represents an independent module, module 13, and several course units in other modules specifically address objectives such as sensitive care, focus on relationships during care of infants and close collaboration with families.

## Didactic approach of the ESN MAS curriculum

The ETR Neonatology provides a standardized set of minimum training requirements for neonatology, including a comprehensive set of guidelines for successful neonatology training programs, which includes goals, timelines, requirements, skills, and competencies. To put these guidelines and requirements into practice, a neonatology training program must meet the needs of the unique professional circumstances of pediatricians and qualified pediatric nurses interested in pursuing a subspecialty in neonatology.

A successful neonatology training program must provide the necessary skills and knowledge in a way that matches the time and resources available for busy professionals in a variety of different contexts. One approach that has found success in neonatology training is online education^7,8^ (Hall et al.^6^). While challenges exist in facilitating interaction, online training modules provide flexibility for time use and the knowledge provided can then be used in the learner’s individual context^8^ (Hall and Smith^7^). The ESN MAS in Neonatology curriculum incorporates online training modules, which allows for asynchronous and distance learning that can fit trainees’ schedules.

Another training approach within the ESN MAS in Neonatology curriculum is the use of Entrustable Professional Activities (EPAs) and Transfer into Practice (TIP) tasks to assess the readiness of trainees for independent practice.^26^ EPAs are tasks or groups of tasks that healthcare providers must be able to perform independently and safely, which are based on the core competencies and skills needed for medical practice^16^ (Parker et al.^25^). EPAs can be applied to any of the neonatology skills learned during training because they include a rating of entrustability that can help identify the skill level that a learner has attained and is expected to attain (Supplement file).

TIP tasks, on the other hand, provide hands-on training and practice opportunities for trainees to apply their knowledge and skills in real clinical settings, under the supervision of experienced neonatologists. Within the flexible structure of the MAS program, instructors and learners can work together to identify opportunities for TIP tasks to match skill level and local context of the learner. While not explicitly identified within the modules of the curriculum, TIP tasks can be used to practice most of the identified skills.

By combining EPAs and TIP tasks, the ESN MAS curriculum ensures that trainees not only acquire the necessary knowledge, but also develop the practical skills and experience needed for independent practice in neonatology (see ESN MAS didactic objectives in Table 2). Both EPAs and TIPs require assessment by a supervisor at trainees’ work site for clinically relevant evaluation. This creates a structured feedback process between trainees’ local supervisor and MAS program educators, which is a central component of the program, as discussed below.

**Table 2:** ESN MAS curriculum didactic objectives.

Self-directed training	Implementation and purpose
Activation of basic knowledge	Material and tasks are didactically prepared to activate basic knowledge and present new information
Simple mini-cases	Existing knowledge is brought into practice-relevant structure using small and rather simple cases in which a few clinical decisions have to be made.
Decision making in evolving cases	To reflect clinical practice, online training is done step by step, with cases evolving based on trainee decisions.
Interprofessionalism	Many clinical situations are interprofessional in nature and thus require activities of others to be planned, anticipated, or reviewed. Various concepts, stories or tips addressing this are given, useful for beginners and advanced learners.
Adapt Standard Operating Procedures (SOP)	SOPs summarize valid knowledge in common settings. They are part of quality assurance and reflect local context and management. SOPs for specific topics are presented, providing assignments for practice to train adaptation to given situation
Training in practice	Online courses are limited in their methods by nature. Since participants work in clinical practice, these limitations can be partially alleviated when participants receive specific tasks for clinical practice.
Enhance quality management	While students go through the online documents, they find dedicated assignments to be carried out in their own clinic environment in order to increase the quality of their work (continuing education, operating procedures, critical incident management, etc.).
**Tasks with output discussed in the peer and tutor group**	
Action strategies: Own case discussions	Students are asked to present their own anonymized cases from their own clinical work including information on decision making and uncertainties. This is about making your own decisions visible, debatable, and thus changeable. The tutor group provides feedback on these case presentations, rationales, action strategies, which all focus on a specific topic.
Work on beliefs, culture, and context	Students receive small tasks that allow for different approaches or strategies. They write their solutions, which then are discussed in the group reflecting different regional contexts, priorities, social, health settings and clinical cultures, clinical procedural guidelines. All of which focus on a specific topic.
Discussion tasks for tutor groups	In everyday clinical practice, many decisions of technical or medical nature can be subject to debate (i.e. subject to clinical judgment). Give some prompts related to the specific topic for discussion in the tutor peer group.
Communication with other healthcare professionals	Long-decision-making cases contain elements that train communication with other professionals, e.g. for effective and quick internal and external patient handover. Some dos and don’ts are provided, possibly in addition to a training or observation assignment.
Collaboration with specialists	Neonatologists work together with other medical disciplines and specialists, e.g. radiology, cardiology, surgery, microbiology. Some dos and don’ts are provided, possibly in addition to a training or observation assignment.
Cooperation with parents and/or legal patient representatives/guardians	Cases or tasks include training aspects of work with parents that are relevant to the topic. Alternatively or in addition, give a training or observation assignment, for example in child protection.
Management	Management includes work organization, order placement, cost-conscious decision-making, procurement-management, reporting, and more. Give some topic-related concepts, tips, and assignments on these management aspects.

## Evaluation framework

Effective evaluation of competency-based medical education is critical for trainee success.^27^ The ESN MAS in Neonatology curriculum evaluation framework is based on the EPA system, which has five levels of entrustability, ranging from ‘*trusted to observe only’* to ‘*trusted to execute without supervision’*. To evaluate the core components essential to training progress,^28^ each EPA is assessed by their local supervisor, who will receive regular updates from the ESN on the trainees’ performance. Trainees are assessed in five categories, including the management of acute single system diseases, complex multisystem diseases, patients with surgical problems, resuscitation and stabilization, and neonatal care systems. The aim of this evaluation is to ensure that trainees meet the requirements for acquiring the MAS diploma, and that they are ready to practice independently and safely in the field of neonatology. The curriculum includes a detailed framework laying out the skills required for each category of competence, and an EPA level of at least four out of five in each category is needed to successfully complete the program.

Just as trainees in the program will be evaluated on their progress, the curriculum and its implementation are subject to regular quality evaluation. The 13 modules of the ESN MAS are carefully constructed to form a total of 60 course units. Each course unit includes at least eight out of the 14 didactic objectives to ensure that each unit maximizes clinically relevant learning opportunities. To assure that each objective and course unit are effective, they will be assessed regularly throughout program implementation to ensure that they cover the required skills and competencies (see Supplementary Appendix 1 for the complete ESN MAS curriculum). The curriculum itself will undergo periodic internal review to ensure that the contents are aligned to the most up-to-date best practices in neonatology and to ensure that it provides a strong standard for the knowledge and skills of neonatology practitioners.

## Conclusion

The ESN’s MAS in Neonatology curriculum addresses the need for a standardized and comprehensive training program for neonatal care by providing a structured framework that aligns with the European training requirements and incorporates EPAs and TIP tasks for effective assessment of trainees’ readiness for independent practice. The curriculum covers a wide range of topics relevant to neonatal care and includes a robust evaluation framework to ensure that trainees meet the necessary competencies and skills for safe and effective practice and empowering of parents. Implementing the ESN MAS in Neonatology curriculum as the standard for neonatal training and education across Europe can help standardize the quality of neonatal care, ensuring that all neonates receive evidence-based care regardless of their location and improve patient outcomes by reducing the occurrence of adverse events and complications and by empowering parents and families.

While the ESN MAS Neonatology curriculum has the components to meet the training need within Europe, there are elements of neonatology training that must be incorporated into the program during implementation. These elements include providing explicit training in ethics, ward organization, CRM, and providing experiential learning opportunities through reflective practice. While these elements may be contained in other components of the curriculum, they are worthy of individual focus to assure that the practitioners who emerge from European training programs have the highest ethical standards, the skills needed to run an effective team in their individual context and are motivated to continue to improve their practice.

The implementation of the ESN MAS curriculum comes with a set of advantages and challenges. The curriculum can provide a platform for hybrid learning that makes it uniquely flexible to a wide variety of contexts and the needs of novice practitioners and training sites. By offering an international tutor and trainee peer group support and exchange, the MAS program offers opportunities to share ideas and learning and raise international learning standards. However, along with these opportunities, language barriers and differences between learning contexts represent challenges in implementing the MAS curriculum. Implementing the curriculum and providing international training in neonatology will require communication of complex technical information in a variety of languages. Individual training sites may have differences in approaches and resources that create a challenge at making the MAS content broadly applicable and understandable in different languages. These challenges must be considered during implementation of the MAS program by eliciting and applying stakeholder feedback and through open communication to assure that the needs of trainees, training sites, and ultimately of patients are being met successfully.

The strength of the ESN MAS curriculum is in the rigorous standards it sets for neonatal care by incorporating up to date clinical practices and knowledge, aligning with the ETR Neonatology, and drawing on educational best practices. It is uniquely positioned to set an international standard for neonatology training to equip healthcare providers with essential competencies and skills while ensuring safe and effective practice, which will enhance patient outcomes and the overall quality of neonatal care. By bringing the European Training Requirements for Neonatology to practice, the ESN’s curriculum contributes to the professional development of neonatal care providers, ultimately improving the quality of care across Europe and beyond.

## Supplementary Information

Supplement file
