# Supplementary material for: Meeting the need for effective and standardized neonatology training: a pan-European Master’s Curriculum
Source: Pediatr Res. 2024 May 3;96(5):1195–200. doi: 10.1038/s41390-024-03182-8 (PMC11521982; doi:10.1038/s41390-024-03182-8)
Supplement: Supplementary file 1 — Supplement file [file 41390_2024_3182_MOESM1_ESM.pdf]

European School of Neonatology

European Curriculum & Evaluation Grid for Training and Assessment in Neonatology

Version 2, Completed October 14, 2023

Authors: Sven Wellmann, Manfred Künzel

Approved by the Board of the European School of Neonatology (ESN): October 31, 2023

Preface

Founded in 1958, the European Society for Paediatric Research (ESPR) has the mission to develop and apply research to improve newborn and child health. The European School of Neonatology (ESN) represents the educational arm of the ESPR in the paediatric subdiscipline Neonatology. In line with its overarching goal of improving professional neonatal care in Europe and beyond, the ESN has developed an interprofessional master programme offering medical doctors and nurses a tutored supranational training framework in Neonatology, which can be completed with the degree of a Master of Advanced Studies (MAS). The curriculum and evaluation grid are provided here.

Table of contents

1. Introduction

2. Training

  1) Resuscitation and stabilisation

  2) The very and extremely preterm infant

  3) The moderate and late preterm infant

  4) Cardiorespiratory failure

  5) Life-threatening infection

  6) Brain-injured newborn

  7) Nutrition of the critically ill newborn

  8) Surgical problems

  9) Single system diseases

  10) Bronchopulmonary dysplasia

  11) Difficult care conference

  12) Neonatal transport, patient flow management and transition of care

  13) Infant and family centred developmental care

  List of abbreviations

3. Evaluation for clinical training and assessment

  Category 1: Manage patients with acute, common single system diseases in an inpatient setting

  Category 2: Manage patients with complex, multisystem diseases in the NICU

  Category 3: Provide care to patients in the NICU with surgical problems in collaboration with Paediatrics and subspecialty surgeons

  Category 4: Provide resuscitation and stabilisation of neonates and infants that aligns care with severity of illness

  Category 5: Management of neonatal care systems

4. References

1. Introduction

The European Training Requirements (ETR) in Neonatology published in 2021 represents the ESPR, EAP (European Academy of Paediatrics) and UEMS (Union of European Medical Specialists) accredited syllabus for neonatal training in Europe, that has been published in 2021 and serves as the basis of all educational offers of the ESN, including the MAS in Neonatology. The teaching objectives listed in the ETR adhere to the principles of infant and family centred developmental care (IFCDC). The ESN MAS in Neonatology aims i) to teach all ETR key competencies and cross cutting skills and topics in Neonatology (chapter 6.4 and 8 of the ETR Neonatology), ii) to translate theory and evidence behind IFCDC into students practise, and iii) is constructed based on modern professional medical education involving Entrustable Professional Activities (EPAs) and Transfer into Practice (TIP) tasks.

EPAs are a way of assessing the readiness of medical graduates for independent practice. They are defined as a task or a group of tasks that healthcare providers must be able to perform independently and safely. EPAs are based on the core competencies and skills that are needed for medical practice. They are evaluated through direct observation and assessment of a healthcare provider's performance in the clinical setting. EPAs are designed to help assess the degree to which an individual has attained the knowledge, skill, and attitude to practise independently and safely. By contrast, TIPs foster hands-on medical training and practice by defining clinical tasks to be implemented by the trainee at the local hospital or institute under the supervision of a senior neonatologist. This curriculum provides an overview of all competencies taught in the MAS - the training part - as well as an evaluation framework for hands-on clinical-training and assessment.

2. Training

The ESN MAS training is structured in accordance with the competency system presented by Parker et al. (2017). It has been refined by the ESN didactic team and approved by its Board of experts. This competency system provides a training plan with 13 coherent MAS modules subdivided into 60 course units representing specific activities in Neonatology. The curriculum encompasses all key competencies as defined in the ETR Neonatology and provides chapter references as to where to find them. Selected ESN MAS training units are offered by dedicated partner institutions as indicated. The ESN MAS offers in total 60 ECTS credits, out of which up to 20 credit points can be acquired outside the ESN MAS, e.g. in the Neonatal Online Training and Education (NOTE) programme, and may be accredited on request. More details are given in the ESN MAS regulations.

Table 1: Summary of all 13 ESN MAS modules and 60 course units with reference to all respective key competencies described in the ETR Neonatology

MAS Modules	Course Units (Competencies)
**1) Resuscitation and stabilisation**<br>ETR Neonatology<br>6.4.1 Family care and care of the newborn baby<br>6.4.5 Fluid balance, thermoregulation and renal failure<br>6.4.8 Cardiorespiratory intensive care<br>6.4.11 Resuscitation/stabilisation<br>6.4.14 Communication skills and counselling<br>6.4.15 Ethics	**Provides antenatal counselling, resuscitation, and stabilisation of a critically ill newborn.** *Institutes and leads neonatal resuscitation and stabilisation according to the latest European Resuscitation Council (ERC)/International Liaison Committee on Resuscitation (ILCOR) Guidelines and respective national and institutional guidelines.*<br>1. Performs antenatal counselling, knows the leading causes of prematurity and obstetric emergencies and recommends important measures such as antenatal steroids.<br>2. Communicates effectively and appreciatively with team and parents during intrapartum care, collaborates as a member of an interprofessional team and decides on progression or stopping life supporting care in critically compromised patients.<br>3. Enables measures to conduct resuscitation and stabilisation of the infant, performs thermal-management and anticipates the gestational age specific disease spectrum. Knows principles and distinctions between interventions to restore vital organ functions (resuscitation) or to support transition (stabilisation).<br>4. Applies manual skills confidently such as non-invasive ventilation, endotracheal intubation, chest compressions, vascular access, chest drain insertion.
**2) The very and extremely preterm infant**<br>ETR Neonatology<br>6.4.1 Family care and care of the newborn baby<br>6.4.4 Neurology<br>6.4.5 Fluid balance, thermoregulation and renal failure	**Cares for the very preterm (<32 weeks gestation) and extremely preterm infants (<28 weeks gestation).** *Performs transition at the threshold of viability and recognises the risk factors for morbidity and mortality of this highly vulnerable group of neonates.*<br>5. Understands the physiology of transition in the very and extremely preterm infant born at the threshold of viability, including thermoregulation, fluid and sugar hemostasis, and ensures safe transport processes for the complete patient pathway from delivery to final NICU place.

6.4.8 Cardiorespiratory intensive care<br>6.4.11 Resuscitation/stabilisation	6. Provides initial and continuing respiratory support for very and extremely preterm infants.<br>7. Maintains neurologic integrity in the very and extremely preterm infant, including prevention, examination and treatment of common neurologic disorders such as IVH and PVL.<br>8. Manages parental and enteral nutrition, regards latest recommendations on nutrition, and performs measures for prevention, examination and treatment of meconium ileus, FIP, NEC and growth failure.<br>9. Facilitates maintenance in circulation, perfusion and tissue oxygenation immediately after delivery and during NICU stay, manages arterial blood pressure and PDA dynamics.
**3) The moderate and late preterm infant**<br><br>ETR Neonatology<br>6.4.1 Family care and care of the newborn baby<br>6.4.5 Fluid balance, thermoregulation and renal failure<br>6.4.7 Haematology and transfusion<br>6.4.8 Cardiorespiratory intensive care<br>6.4.10 Congenital anomalies and genetic disease<br>6.4.11 Resuscitation/stabilisation	**Cares for the moderate preterm and late preterm infants (≥32 weeks gestation).** *Performs transition and institutes all diagnostic and therapeutic measures in healthy and critically compromised preterm infants.*<br>10. Provides respiratory support for infants with IRDS, TTN, meconium aspiration, PTX and PPHN.<br>11. Examines the newborn baby clinically, recognises injuries associated with the birthing process and common congenital anomalies and dysmorphologies. Formulates a plan for further investigations.<br>12. Recommends and interprets common diagnostic and screening tests according to national and institutional guidelines.<br>13. Manages hematologic emergencies, including bleeding, anaemia and disseminated intravascular coagulation. Knows relevant diagnostic tests, rules of transfusion of blood products and exchange transfusion.<br>14. Prescribes preventive and therapeutic measures for early nutrition and blood sugar maintenance in infants with IUGR, SGA and LGA.<br>15. Manages inpatient and outpatient neonatal jaundice, initiates examination and treatment of indirect hyperbilirubinemia and is able to distinguish between neonatal cholestasis and hepatitis.
**4) Cardiorespiratory failure**<br><br>ETR Neonatology<br>6.4.8 Cardiorespiratory intensive care<br>6.4.10 Congenital anomalies and genetic disease<br>6.4.11 Resuscitation/ stabilisation<br>governance<br>6.4.15 Ethics	**Cares for infants with cardiorespiratory failure.** *Manages emergencies resulting from cardiac or respiratory causes. Understands the pathophysiology of neonatal circulation and neonatal breathing and recognises specific diseases.*<br>16. Institutes and maintains full cardiorespiratory intensive care for neonates, enabling organ perfusion and oxygenation following latest guidelines on fluid and drug prescription.<br>17. Recognises life threatening heart defects, including ductal dependent lesions, profound shock and congestive heart failure, formulates a detailed care plan and institutes appropriate initial care.<br>18. Recognises respiratory emergencies, including congenital anomalies of the lung such as CDH, CPAM and congenital emphysema, formulates a detailed care plan and institutes appropriate initial care.<br>19. Recognises and manages abnormal heart rhythm, including tachyarrhythmias and bradyarrhythmias such as LQT syndrome.

	20. Manages non-invasive ventilation support, knows the principles and application of a range of non-invasive modalities for acute and chronic lung disease and assesses the need for therapy escalation in line with gestational age.<br>21. Manages invasive ventilation strategies, knows the principles and application of a range of invasive modalities for acute and chronic lung disease and assesses the need for therapy de-escalation in line with gestational age.<br>22. Performs basic point-of-care echocardiography (ECHO, e.g. NPE basic course).
**5) Life-threatening infection**<br>ETR Neonatology<br>6.4.3 Immunity and infection<br>6.4.14 Communication skills and counselling	**Cares for infants with life-threatening infection**. *Knows principles of pathophysiology, pathogenesis and clinical presentation of neonatal infections. Performs physical examination and recommends diagnostic testing.*<br>23. Performs antenatal counselling for vertical transmittable diseases transplacental, intrapartum and postpartum (TORCH, HBV, HIV, SARS-Cov2). Knows national recommendations for hygiene measures, vaccination and initial newborn care.<br>24. Knows principles of the developing immunity, infection control, treatment of its compromise and preventive measures such as active and passive vaccinations in accordance with latest recommendations.<br>25. Recognises signs and symptoms of local and systemic infections and compromised immunity, investigates and develops care plans for viral, bacterial and fungal infections.<br>26. Manages infections of specific organ systems, including respiratory infections (RSV bronchiolitis, pneumonia and pertussis), urinary tract infections, septic arthritis and osteomyelitis.<br>27. Contributes to a culture of hygiene and antimicrobial stewardship.
**6) Brain-injured newborn**<br>ETR Neonatology<br>6.4.4 Neurology<br>6.4.14 Communication skills and counselling<br>6.4.15 Ethic	**Cares for the brain-injured newborn.** *Performs clinical examinations, assesses integrity of the brain using neurophysiologic and imaging tools, initiates therapy and recommends post discharge care and follow-ups of high-risk neonates.*<br>28. Identifies abnormal neurological behaviour, prioritises differential diagnosis of the crying, jittery, hypotonic neonate or the neonate presenting with seizures based on clinical evaluation. Recommends and interprets common diagnostic tests of neurophysiology, brain imaging and tests of serum and CSF and prescribes initial cause-specific therapy.<br>29. Manages infants with asphyxia, grades HIE and initiates neuroprotective measures. Understands distinction to PAIS, SVT or other diseases.<br>30. Forms a supportive post-discharge management plan for neurodevelopmental follow-ups and rehabilitative therapies, in close collaboration with families, involving specialists and therapists, respecting regional health-care structures.<br>31. Performs basic cerebral ultrasound (cUS).

**7) Nutrition of the critically ill newborn**<br><br>ETR Neonatology<br>6.4.5 Fluid balance, thermoregulation and renal failure<br>6.4.6 Nutrition, feeding, gastro-intestinal and hepatic disease<br>6.4.10 Congenital anomalies and genetic disease	**Manages nutritional needs of critically ill newborns.** *Cares for neonates with gastrointestinal emergencies and collaborates interprofessionally with paediatric radiologists and paediatric surgeons to advance patient care.*<br>32. Assesses nutritional requirements, knows principles of standardised parenteral nutrition and formulates a balanced feeding plan in a gestational age specific manner.<br>33. Recognises and distinguishes non emergent from emergent causes in neonates with vomiting, diarrhoea, constipation and gastrointestinal bleeding, initiates appropriate diagnostics and initiates cause-specific care.<br>34. Recognises and manages common congenital gastrointestinal and hepatic anomalies.<br>35. Forms a post-discharge management plan in close collaboration with families, involving specialists and therapists, respecting regional health-care support structures including feeding support modalities for home care and knows the risk factors for development of infant eating disorders and severe malnutrition in later life.
**8) Surgical problems**<br>ETR Neonatology<br>6.4.1 Family care and care of the newborn baby<br>6.4.5 Fluid balance, thermoregulation and renal failure<br>6.4.10 Congenital anomalies and genetic disease:<br>6.4.13 Structured clinical hand over	**Provides care to patients in the NICU with surgical problems in collaboration with paediatric and subspecialty surgeons.**<br>36. Provides preoperative and postoperative care including sedation and analgesia to infants in the NICU with surgical problems.<br>37. Performs antenatal counselling, establishes a disease-specific management plan for delivery and performs neonatal management acquired acute and chronic abdominal disorders such as volvulus, gastrointestinal atresia, Hirschsprung disease, meconium ileus, anorectal malformations, omphalocele or gastroschisis, inguinal hernia.<br>38. Performs antenatal counselling, establishes a disease-specific management plan for delivery and performs neonatal management of genitourinary emergencies, including renal abnormalities, collecting system and bladder abnormalities, penile and urethral abnormalities, scrotal abnormalities, ambiguous genitalia, gynecologic abnormalities.<br>39. Performs antenatal counselling, establishes a disease-specific management plan for delivery and performs neonatal management of orthopaedic emergencies, including nonaccidental trauma, fractures, brachial plexus injuries, muscular torticollis, developmental hip dysplasia.<br>40. Performs antenatal counselling, establishes a disease-specific management plan for delivery and performs neonatal management of neural tube defects and congenital hydrocephalus.
**9) Single system diseases**<br><br>ETR Neonatology<br>6.4.4 Neurology<br>6.4.6 Nutrition, feeding, gastro-intestinal and hepatic disease	**Manages patients with acute common single system diseases in an inpatient setting.**<br>41. Retinopathy of prematurity (ROP).<br>42. Pathological hearing test, congenital and acquired deafness.<br>43. Neonatal abstinence syndrome (NAS).

6.4.9 Metabolism and endocrine disorders<br>6.4.10 Congenital anomalies and genetic disease	44. Dermatologic disorders in the first days of life, including birthmarks, hemangiomas, transient vascular phenomena, transient skin eruptions and infectious conditions of the skin, infectious conditions of the skin and cutaneous manifestations of systemic diseases.<br>45. Congenital metabolic and endocrine emergencies.
**10) Broncho pulmonary dysplasia**<br>ETR Neonatology<br>6.4.3 Immunity and infection<br>6.4.5 Fluid balance, thermoregulation and renal failure<br>6.4.8 Cardiorespiratory intensive care	**Cares for infants at risk and with established bronchopulmonary dysplasia.**<br>46. Knows epidemiology and preventive strategies of BPD.<br>47. Performs diagnostic studies of BPD and manages infants with all stages of BPD including cardiac remodelling and pulmonary hypertension.<br>48. Forms a post-discharge management plan in close collaboration with families, involving specialists and therapists, respecting regional health-care support structures including respiratory support modalities for home care and knows the risk factors for development of chronic obstructive pulmonary disease (COPD) in later life.
**11) Difficult care conference**<br>ETR Neonatology<br>6.4.1 Family care and care of the newborn baby<br>6.4.12 Ward organisation / Management skills / Clinical governance<br>6.4.14 Communication skills and counselling<br>6.4.15 Ethics	**Leads difficult care conferences, including discontinuation of life support**.<br>*Identifies system failures and contributes to a culture of safety and improvement.*<br>49. Manages difficult medical and emotional settings with empathy, maintains a critical incident reporting system, adheres to principles of medicolegal responsibilities and importance of documentation.<br>50. Obtains informed consent from parents for tests or procedures, checks for organ donation and autopsy.<br>51. Knows the basic ethics of newborn medicine, including recognition and practice of treatment at the threshold of viability and end-of-life care decisions in accordance with the national law. Uses empathy when communicating redirection of care and is trained in shared decision-making to be able to embark in such processes with the obstetrical, neonatal teams and with the parents.
**12) Neonatal transport, patient flow management and transition of care**<br>ETR Neonatology<br>6.4.1 Family care and care of the newborn baby<br>6.4.2 Transport of the newborn baby<br>6.4.5 Fluid balance, thermoregulation and renal failure<br>6.4.13 Structured clinical hand over<br>6.4.12 Ward organisation / Management skills / Clinical governance	**Coordinates transport of critically ill newborns, manages transition of care: routine sign-out, change of service.**<br>52. Prepares quickly and efficiently for taking over patient responsibility by gathering patient history, performing clinical exams and checking technical installation and equipment.<br>53. Manages technical breakdowns and unexpected clinical emergencies out of hospital.<br>54. Practices a culture of briefing and debriefing of extremely stressful situations, employs techniques of mental and physical self-care.<br>55. Manages patient flow on the NICU with admissions and retrieval.<br>56. Gives or receives a patient handover to transition care responsibility, performs information and documentation efficiently, medico-legally sufficiently and in line with data protection regulations.
**13) Infant and family centred developmental care**<br>ETR Neonatology	**Cares for infants and families in a development-oriented environment, has the long term outcome of the infant in mind.**<br>*Supports a culture of involving parents and adheres to the standards for infant- and family-centred developmental care.*

6.4.1 Family care and care of the newborn baby<br>6.4.13 Structured clinical hand over<br>6.4.14 Communication skills and counselling	57. Adheres to the basic principles of IFCDC, including sensitive care, mindfulness of the relationships between infants, families and professionals, and individualised care (FINE level 1 course).<br>58. Enables a supportive environment of care with limited stress exposure, optimal sensory ambience and maturity appropriate motoric stimulation with family involvement.<br>59. Knows and adheres the principles of lactation, breastfeeding and kangaroo care, and fosters infants nurture and parent-infant bonding.<br>60. Practices discharge home of infants, develops an individual case management plan, organises needed investigations, procedures and follow-ups in close collaboration with families and ensures the competencies and resources of families prior to discharge.

List of abbreviations

BPD Bronchopulmonary Dysplasia
CDH Congenital Diaphragmatic Hernia
COPD Chronic obstructive pulmonary disease
CPAM Congenital Pulmonary Airway Malformation
CSF CerebroSpinal Fluid
cUS Cerebral Ultrasound
ECTS European Credit Transfer and Accumulation System
ERC European Resuscitation Council
FINE Family and Infant Neurodevelopment Education
FIP Focal intestine perforation
HBV Hepatitis B-Virus
HIE Hypoxic Ischemic Encephalopathy
HIV Human Immunodeficiency Virus
FIP Focal intestine perforation
IFCDC Infant and family centred developmental care
ILCOR Int. Liaison Committee on Resuscitation
IRDS Infant Respiratory Distress Syndrome
IUGR Intrauterine growth restriction
IVH Intraventricular Haemorrhage
LGA Large for gestational age
LQT Long-QT syndrome
NAS Neonatal Abstinence Syndrome
NEC Necrotizing Enterocolitis
NOTE Neonatal Online Training and Education
NICU Neonatal Intensive Care Unit
NPE Neonatal Performed Echocardiography
PAIS Perinatal Arterial Ischemic Stroke
PDA Patent Ductus Arteriosus
PPHN Persistent Newborn Pulmonary Hyperten.
PTX Pneumothorax
PVL Periventricular Leukomalacia
ROP Retinopathy of prematurity
RSV Respiratory syncytial virus
SARS-CoV2 Severe Acute Respiratory Syndrome Coronavirus 2
SGA Small for gestational age
SVT Sinus Venosus Thrombosis
TORCH Toxoplasmosis, others, Rubella, Cytomegaly, Herpes
TTN Transient Tachypnea of the Newborn

3. Evaluation for clinical training and assessment

For the evaluation of the trainees' learning progress within the MAS in Neonatology, the ESN has chosen the EPA system as established by the American Board of Pediatrics (ABP) and adapted it for its purposes. The EPA system enables the assessment of the outcomes of the MAS training combined with TIP-based clinical training and work.

The EPA system in general has five levels of entrustability (American Board of Pediatrics, n.d.):

1. Trusted to observe only;
2. Trusted to execute with direct supervision and coaching;
3. Trusted to execute with indirect supervision for most simple and some complex cases;
4. Trusted to execute with indirect supervision but may require discussion of information conveyed for a few complex cases;
5. Trusted to execute without supervision.
   5a. Not yet ready to also supervise others in the execution of this EPA
   5b. Also trusted to supervise others in the execution of this EPA

The level of entrustability will be checked during the MAS training and after its completion together with the trainees' local supervisors. The aim of this EPA-based evaluation is thus to determine the trainees' progress and eventually check whether all requirements for acquiring the MAS diploma have been met. There are 5 categories in which the levels of entrustability will be assessed, as described below. Whereas categories 1 to 4 refer to medical competencies trained in various modules and course units, category 5 is limited to organisational and management competencies, which are trained in module 13. In detail, the local supervisor will receive online documents including the 5 categories from the ESN on a regular basis in order to rate the trainees' progress in terms of entrustability. The ESN expects an EPA level of at least 4 on average in all 5 categories for passing the final check.

Category 1: Manage patients with acute, common single system diseases in an inpatient setting

Neonatologists must be able to provide care for neonates and infants who have a broad range of illnesses, including those that predominantly affect a single organ system. Care of these patients must take into consideration relevant pregnancy and delivery circumstances and the gestational age of the infant. Common, single-system diseases can evolve to include multiple systems and greater complexity. Neonatologists must anticipate these potential complications and recognise when they occur. It is beyond the scope of this document to attempt to identify all of the single system diseases for which a neonatologist would be responsible; rather the document includes illustrative problems that a practising neonatologist will see commonly.

Evaluation framework:

1. Gathering essential information through history taking, physical exam, and judicious laboratory evaluation.
2. Using sound clinical reasoning to develop a prioritised differential diagnosis that allows the proper diagnostic tests to be performed.
3. Knowing or acquiring knowledge of the evidence related to the primary problem, taking gestational age into consideration as appropriate.

4. Applying the evidence to the patient's care in developing a management plan that addresses the primary problem, the need for hospitalisation, plans for nutritional support, discharge criteria, and follow-up plans.
5. Placing the patient at the centre of all management decisions to provide patient and family centred care by engaging in bidirectional communication with caregivers and parents.
6. Disseminating the therapeutic plan and clinical reasoning in a manner that is transparent to all members of the healthcare team.

Category 2: Manage patients with complex, multisystem diseases in the NICU

Neonatologists must be able to provide care for neonates and infants with a broad range of illness, including those who present with complex illnesses involving multiple systems and those who deteriorate after presenting with common, single-system diseases. Although most newborns requiring the care of a neonatologist will present at birth, in some circumstances, neonatologists may be expected to provide care to infants who have previously been well and at home.

Evaluation framework:

1. Developing a complex management plan to provide care to infants with a broad range of complex diseases that affect multiple organ systems. In addition to addressing and documenting the primary problem, documentation of admission and discharge criteria, and nutritional plans, the management plan must also address all comorbidities (actual and reasonably anticipated) requiring attention during the entire hospitalisation, and case management issues such as special resources required during and after hospitalisation.
2. Coordinating care with the interdisciplinary health care team. The complex neonate and infant in the inpatient setting requires the input of the many professionals on the healthcare team (e.g., nutritionist, pharmacist, advanced practitioner, discharge planner, social worker, other subspecialists); thus, the entrusted professional must be able to communicate and partner with both interdisciplinary and intradisciplinary professionals and parents. This activity includes reconciling disparate opinions from other health care professionals and coordinating and prioritising their input.
3. Managing uncertainties (personal, team, and family) is a critical skill since much of the care for these patients is not known or determined clearly in the existing medical evidence.
4. Delivering bad news. Unforeseen errors, life-threatening complications, and poor outcomes are more common in neonates and infants with acute, complex, multi-system disease, and the learner must be entrusted to communicate effectively and compassionately with families.

Category 3: Provide care to patients in the NICU with surgical problems in collaboration with Paediatrics and subspecialty surgeons

Some neonates and infants are born with or develop conditions after birth for which surgery is curative or palliative. Neonatologists must be able to anticipate delivery room needs of infants with known congenital anomalies, as well as identify patients who develop conditions that may benefit from surgery. Optimal pre- and post- operative stabilisation, management, and communication help ensure optimal outcomes.

Evaluation framework:

1. Recognising conditions where surgery is indicated primarily or in conjunction with medical management, including knowledge of the limitations of one's ability to manage problems medically.
2. Providing management and/or stabilisation pre- and post-operatively of neonates and infants with problems requiring surgical intervention to include appropriate nutritional, cardiopulmonary, and pain management.
3. Communicating directly with the paediatric general or subspecialty surgeon to facilitate the timely and coordinated care of neonates and infants with surgical needs.
4. Providing continuity of care that ensures that parents understand the diagnosis, management, and follow-up needed and is able to take shared decisions.

Category 4: Provide resuscitation and stabilisation of neonates and infants that aligns care with severity of illness

Ten percent of newborns require some assistance to breathe at the time of birth, and 1% will require extensive cardiopulmonary resuscitation to survive. After birth, many neonates admitted to the neonatal intensive care unit (NICU) will require cardiopulmonary resuscitation at some point during their hospital stay. Therefore, neonatologists must be able to provide resuscitation and stabilisation of neonates and infants that aligns care with severity of illness.

Evaluation framework:

1. Anticipating and recognising the sick neonate and infant requiring resuscitation. This activity includes providing effective counselling to parents with a foetus at risk, both before and after delivery.
2. Prioritising and implementing management in an expeditious fashion, particularly when procedures such as airway management and vascular access are indicated.
3. Effectively leading an interprofessional team before, during, and after resuscitation. Reflecting on one's own behaviours and providing feedback to other learners to improve future resuscitations.

Category 5: Management of neonatal care systems

Neonatologists must be able to anticipate and manage the needs of the entire NICU population to maximise safe and effective care for individual patients.

Evaluation framework:

1. Fluidly assessing the needs of a NICU population with changing acuity and census to optimise outcomes for all.
2. Prioritising deployment of resources (transport, beds, nursing, front line clinicians, etc.) to those patients with the highest acuity and/or most pressing needs.
3. Developing a utilisation plan that incorporates the prioritised needs of individual patients, the composition, and capabilities of personnel with the care environment (NICU, transport, delivery room, OR, radiology suite, etc.), and the needs of parents and family support.

4. Delegating responsibilities appropriately to other qualified members of the care team when necessary.
5. Recognising limits and asking for help.
6. Communicating utilisation and deployment plans to personnel in a succinct and timely manner that contributes to the effectiveness of the utilisation plan.
7. Anticipating the potential for sudden changes to the NICU system and developing contingency plans to account for and adapt to predictable changes in acuity and/or census.
8. Creating a unit culture involving parents.

4. References

American Board of Pediatrics. (n.d.). *Entrustable Professional Activities for General Pediatrics*. Retrieved 15.12.2022, from https://www.abp.org/content/entrustable-professional-activities-general-pediatrics

Parker, T. A., Guiton, G., & Jones, M. D., Jr (2017). Choosing entrustable professional activities for neonatology: a Delphi study. *Journal of perinatology : official journal of the California Perinatal Association*, *37*(12), 1335–1340. https://doi.org/10.1038/jp.2017.144

Wellmann S, Künzel M, Fentsch P, Fauchère JC, Rabe H, Szczapa T, Dimitriou G, Vento M, Roehr CC. Opinion Paper: Rationale for Supra-National Training in Neonatology. Front Pediatr. 2022 Jul 1;10:899160. doi: 10.3389/fped.2022.899160. PMID: 35844736; PMCID: PMC9283761.
